# Alteration of plasma metabolic profile and physical performance combined with metabolites is more sensitive to early screening for mild cognitive impairment

**DOI:** 10.3389/fnagi.2022.951146

**Published:** 2022-07-26

**Authors:** Yinjiao Zhao, Peiyu Song, Hui Zhang, Xiaoyu Chen, Peipei Han, Xing Yu, Chenghu Fang, Fandi Xie, Qi Guo

**Affiliations:** ^1^Jiangwan Hospital of Shanghai Hongkou District, Shanghai University of Medicine and Health Science Affiliated First Rehabilitation Hospital, Shanghai, China; ^2^Department of Rehabilitation Medicine, Shanghai University of Medicine and Health Sciences, Shanghai, China

**Keywords:** mild cognitive impairment, untargeted metabolomics, sphingolipid metabolism, physical performance, phosphatidylcholine

## Abstract

**Objective:**

Unbiased metabolic profiling has been initiated to identify novel metabolites. However, it remains a challenge to define reliable biomarkers for rapid and accurate diagnosis of mild cognitive impairment (MCI). Our study aimed to evaluate the association of serum metabolites with MCI, attempting to find new biomarkers and combination models that are distinct for MCI.

**Methods:**

A total of 380 participants were recruited (mean age: 72.5 ± 5.19 years). We performed an untargeted metabolomics analysis on older adults who underwent the Mini-Mental State Examination (MMSE), the Instrumental Activities of Daily Living (IADL), and physical performance tests such as hand grip, Timed Up and Go Test (TUGT), and walking speed. Orthogonal partial least squares discriminant analysis (OPLS-DA) and heat map were utilized to distinguish the metabolites that differ between groups.

**Results:**

Among all the subjects, 47 subjects were diagnosed with MCI, and methods based on the propensity score are used to match the MCI group with the normal control (NC) group (*n* = 47). The final analytic sample comprised 94 participants (mean age: 75.2 years). The data process from the metabolic profiles identified 1,008 metabolites. A cluster and pathway enrichment analysis showed that sphingolipid metabolism is involved in the development of MCI. Combination of metabolite panel and physical performance were significantly increased discriminating abilities on MCI than a single physical performance test [model 1: the area under the curve (AUC) = 0.863; model 2: AUC = 0.886; and model 3: AUC = 0.870, *P* < 0.001].

**Conclusion:**

In our study, untargeted metabolomics was used to detect the disturbance of metabolism that occurs in MCI. Physical performance tests combined with phosphatidylcholines (PCs) showed good utility in discriminating between NC and MCI, which is meaningful for the early diagnosis of MCI.

## Introduction

Mild cognitive impairment (MCI) is the most prevailing neurodegenerative disease, affecting approximately 15.5% of the population above 60 years (Jia et al., 2020). It is considered a transition phase between normal cognitive aging and early dementia and the state is a “window” in which it may be possible to intervene and delay the progression to dementia (Mufson et al., 2012). Early detection of those most at risk is necessary to properly target early treatment and the current diagnosis of MCI essentially relies on PET neuroimaging and cerebrospinal fluid (CSF) proteins, but these detections are expensive and invasive. Neurobiological features of cognitive impairment are hypometabolism in temporoparietal cortices (Anderson, 2019); therefore, many studies increasingly emphasize the biomarkers that may assist the diagnosis of MCI.

Accumulated evidence indicated that MCI is multifactorial; a combination of age, genetics, and environmental factors might contribute to its onset and progression (Jia et al., 2020). Emerging evidence indicated that peripheral alterations including metabolic dysregulations might precede and contribute to neurodegeneration (Nho et al., 2019; Weng et al., 2019). Liquid chromatography-mass spectrometry (LC-MS)-based metabolomics is the systemic study of small-molecule metabolite profiles of organisms in a certain state or phenotype (Kim et al., 2021). By using metabolomics, it is possible to discriminate antecedent cognitive impairment before the onset of overt clinical symptoms and epidemiological evidence supported a link between metabolic disorders and cognitive impairment (Wang et al., 2020). Several studies have investigated the role of metabolomics in MCI, Alzheimer's disease (AD), and cognitive decline and suggest that cognitive function is closely accompanied by abnormal bile acid metabolism (Mahmoudian Dehkordi et al., 2019; Nho et al., 2019), free fatty acid metabolism (Snowden et al., 2017; Toledo et al., 2017), and lipid metabolism (Han et al., 2011; Varma et al., 2018). However, there are still considerable challenges for blood-based biomarkers, which include a lack of assay development. In this study, we aimed to utilize an advanced high-resolution mass spectrometry metabolomics method capable of capturing the maximum number of spectral features in human plasma.

Mild cognitive impairment (MCI) is diagnosed based on subjective complaints of memory loss or impairment by brief cognitive or neuropsychological tests, and unchanged basic daily functioning (Tricco et al., 2012). Cognitive function is related to the prefrontal cortex, the parietal lobe, and the hippocampus, which affect the physical performance of individuals (Allali et al., 2014), and our previous study has found that physical performance is strongly associated with MCI in older adults (Liu et al., 2021). However, using these tests alone is not sufficient for the differential diagnosis of MCI. Herein, we performed metabolite alteration analysis of plasma in relation to MCI from older adults *via* LC-MS-based untargeted metabolomic technology and established multivariate models with high specificity and sensitivity to accurately differentiate the MCI groups.

## Materials and methods

### Study participants

The research population included residents aged ≥65 years from Shanghai, China, who had joined China's national free physical examination program. A total of 380 subjects were invited to complete a comprehensive geriatric assessment and face-to-face interviewing in the local community hospital. This study was carried out in accordance with the principles of the Declaration of Helsinki. All the subjects provided informed consent before participation.

### Measures of physical performance

All the participants underwent physical examination, detailed neurocognitive assessment, and collection of blood samples. The performance-based assessment consisted of grip strength, Timed Up and Go Test (TUGT), and a 4-m walking test. Grip strength was measured using a dynamometer. Participants were allowed to exert maximum efforts twice using the dominant hand; then, the average value was calculated and adjusted with body weight to ensure muscle strength evaluated was independent of body size. TUGT assessed the seconds of standing up from a chair, walking 3 m at the usual pace past a line on the floor, turning around, walking back to the chair, and sitting down on a chair. The walking speed test consists of participants being timed while walking 4 m at their usual pace, and they were allowed to use a gait assistance device. Participants completed the test two times, and the mean gait speed (m/s) was calculated. The detailed test methods have been described in our previous studies (Song et al., 2021).

### Measures of mild cognitive impairment

The assessment of MCI was based on our previous study (Chen et al., 2021; Liu et al., 2021; Song et al., 2022). The criteria include: (1) memory complaint by patient, family, or physician; (2) normal activities of daily living; and (3) absence of dementia. The classification of MCI and non-MCI was assessed by the Mini-Mental State Examination (MMSE) and the Instrumental Activities of Daily Living (IADL) scale (Su et al., 2014). The cutoff points used for cognitive impairment are as follows: ≤17 for illiterate people, ≤20 for people in primary school, and p 24 for people in middle school or higher (Katzman et al., 1988). The IADL includes 8 items, and the score ranges from 0 to 8 points, with the higher scores indicating better daily living ability. The IADL score of ≥6 indicates intact or only mildly impaired daily living ability (Lawton and Brody, 1969).

### Sample collection and processing

Among the 380 subjects, 333 were normal older adults and 47 were patients with MCI. We obtained 47 normal control (NC) subjects matched by age, sex, body mass index (BMI), and education using propensity score matching. Plasma was collected in EDTA tubes from individuals after an overnight fast using standard venipuncture procedures and then separated and stored at −80°C until analyses. Before the metabolomics analysis, 150 μl plasma was added to a new tube with 10 μl of L-2-chlorophenylalanine (0.3 mg/ml) dissolved in methanol as internal standard, and then 450 μl mixture of methanol/acetonitrile (2/1) was added and vortexed for 1 min. The whole samples were extracted by ultrasonic for 10 min and stored at −20°C for 30 min and then centrifuged at 13,000 rpm for 10 min at 4°C. A total of 200 μl of supernatant was dried in a freeze concentration centrifugal dryer and resolubilized by 300 μl methanol/water (1/4), then vortexed for 30 s, and extracted by ultrasonic for 3 min. After vigorous mixing, samples were centrifuged at 4°C (13,000 rpm) for 10 min and 150 μl supernatants were filtered through 0.22 μm microfilters and transferred to liquid chromatography (LC) vials.

### Untargeted liquid chromatography-mass spectrometry analysis

The analytical instrument was a liquid mass spectrometer system consisting of an ACQUITY ultra-performance liquid chromatography (UPLC) I-Class tandem VION IMS Q-Tof high-resolution mass spectrometer (Waters Corporation, Milford, USA). The samples were separated on the ACQUITY UPLC BEH C18 column (Waters Corporation; 1.7 μm, 100 × 2.1 mm) at a flow rate of 0.4 ml/min. The column was maintained at 45°C, the sample chamber was set at 4°C, and the injection volume was set to 1 μl. The mobile phases were water containing 0.1% formic acid (solution A) and acetonitrile/methanol (2/3, vol/vol) containing 0.1% formic acid (solution B). The gradient was 0–1 min, 30% B; 1–2.5 min, 30–60% B; 2.5–6.5 min, 60–90% B; 6.5–8.5 min, 90–100% B; 8.5–10.7 min, 100% B; 10.7–10.8 min, 100–1% B, 10.8–13 min, 1% B. The ion source was electrospray ionization (ESI) and the sample mass spectrometry signal acquisition was performed in positive and negative ion scanning mode, respectively. Mass spectrometric tuning parameters for LC-MS analysis employed optimized settings as follows: ion source temperature, 150°C; capillary voltages, 2.5 kV; desolvation gas flow, 900 L/h; declustering potential, 40 V; collision energy, 4 eV; mass scan range, m/z 50–1,000; and scan time, 0.2 s.

### Statistical analyses

We used the software Progenesis QI version 2.3 (Nonlinear, Dynamics, Newcastle, UK) for meaningful data mining, performing peak alignment, picking, normalization, and retention time (RT) correction. The resulting matrix of features included information on the mass-to-charge ratio (m/z), RT, and peak intensities. Metabolites were identified by Progenesis QI data processing software based on precise m/z, secondary fragments, and isotopic distribution using the Human Metabolome Database (HMDB) (http://www.hmdb.ca/), lipid maps (version 2.3) (http://www.lipidmaps.org/), METLIN (http://metlin.scripps.edu/), and self-built databases (EMDB) to do qualitative analysis. Comprehensive procedures, including precursor ion alignment and ion fusion, database searching, and scoring, were applied to remove the artifacts and background noise (Zhao et al., 2018). A total of 1,008 compound identifications were automatically linked to the compounds. Orthogonal partial least squares discriminant analysis (OPLS-DA) was performed on the identified metabolites after filtering to distinguish the differences in metabolic profiles between the control and MCI groups. A total of 200 response permutation testing (RPT), including parameters, such as R^2^ and Q^2^, were conducted to assess the quality and reliability of established models. The model is considered an excellent fitness and predictive capability when these parameters are close to 1.0. Differential metabolites between groups were selected using a multidimensional couple with single-dimensional analysis. The variable importance in projection (VIP) generated in OPLS-DA analysis represented differential metabolites with biological significance. Furthermore, the significance of differential metabolites was further verified by Student's *t*-test. Variables with the VIP > 1.0 and *P* < 0.05 were considered to be differential metabolites.

To reveal the mechanism of metabolic pathway variation in different samples, the differential metabolites were carried out with metabolic pathway enrichment analysis based on the Kyoto Encyclopedia of Genes and Genomes (KEGG) database (http://www.kegg.jp/kegg/pathway.html). Their KEGG ID and pathway were found, and then the number of metabolites enriched in the corresponding pathway was calculated. The pathway with a *p* ≤ 0.05 was selected as an enriched pathway; its calculation formula is given as:


$$\begin{array}{l} P=1-\overset{m-1}{\underset{i\;=\;0}{\sum }}\frac{\left(\frac{M}{i}\right)\left(\frac{N-M}{n-i}\right)}{\frac{N}{n}} \end{array}$$

where, N: the total number of metabolites, n: the number of differential metabolites, M: the number of metabolites annotated as a specific pathway, and m: the number of differential metabolites annotated as a specific pathway.

Baseline sociodemographic characteristics between the NC and MCI groups used an independent *t*-test for numerical variables and the chi-squared test for categorical variables. Data with a normal distribution were expressed as the mean ± SD and categorical variables were expressed as proportions. Statistical analyses were performed using SPSS version 26.0 (SPSS Incorporation, Chicago, IL, USA), and *P* < 0.05 was considered statistically significant. The predictive performance of the model was assessed by estimating the area under the receiver operating characteristic (ROC) curve (AUC). We further determined all the significant functional models toward MCI using C-statistics to determine whether combined models improved discrimination ability. The comparison of the area under the ROC curve (AUC) was performed using the MedCalc version 20.0.4 software.

## Results

### Characteristics of the study population

Among the 380 participants (161 men) who were available to be analyzed, 47 (12.4%) met the diagnostic criteria and were defined as having MCI. We matched the MCI group (*n* = 47) with the normal control (NC) group (*n* = 47) in case the differences in metabolic levels owing to age, gender, degree of education, and disease conditions. Totally, 94 participants (mean age 75.2 years, age range 66–87 years) had complete clinical and metabolomics data. Table 1 displays the demographic and clinical characteristics of the participants. Compared with the NC group, participants with MCI had lower physical performance (slower walking speed and poorer mobility) and a lower MMSE score (*P* < 0.05). There was no significant difference in age distribution, gender composition, and the remaining indicators between the groups, which indicated that the subjects in each group were comparable.

**Table 1:** Baseline sociodemographic variables of the matched groups (*N* = 94).

**Characteristic**	**NC (*n* = 47)**	**MCI (*n* = 47)**	***P* value**
Age (years)	74.85 ± 5.09	75.64 ± 5.83	0.488
Sex			0.658
Male (%)	16 (34.04)	14 (29.79)	
Female (%)	31 (65.96)	33 (70.21)	
BMI (kg/m^2^)	24.62 ± 4.13	24.21 ± 3.42	0.598
Education level (%)			0.826
Illiteracy	18 (38.30)	19 (40.42)	
Primary school	22 (46.81)	23 (48.94)	
Secondary school	7 (14.89)	5 (10.64)	
Smoking	4 (8.5)	3 (6.4)	0.694
Drinking	12 (25.5)	13 (27.7)	0.815
Living alone	38 (80.9)	34 (72.3)	0.330
Grip/weight	0.33 ± 0.15	0.33 ± 0.11	0.896
Walking speed	1.08 ± 0.28	0.89 ± 0.26	0.001
TUGT	11.38 ± 3.87	14.23 ± 6.61	0.013
MMSE	23.89 ± 3.14	16.66 ± 3.52	<0.001
Sleep duration	8.76 ± 1.26	8.82 ± 1.68	0.859
IPAQ	5,162.2 ± 4,663.2	4,646.5 ± 5,133.7	0.611
Total cholesterol	5.21 ± 1.13	5.27 ± 1.16	0.798
Triglycerides	1.29 ± 0.63	1.40 ± 0.75	0.433
HDL	1.46 ± 0.37	1.44 ± 0.41	0.842
LDL	3.35 ± 0.92	3.34 ± 0.96	0.960
Number of diseases			
Diabetes (%)	13 (27.7)	13 (27.7)	1.000
Hypertension (%)	29 (61.7)	37 (78.7)	0.071
Hyperlipidemia (%)	9 (19.1)	9 (19.1)	1.000
Stroke (%)	22 (46.8)	20 (42.6)	0.678
Heart disease (%)	20 (42.6)	25 (53.2)	0.302
Osteoarthritis (%)	9 (19.1)	12 (25.5)	0.458
Peptic ulcer (%)	9 (19.1)	6 (12.8)	0.398

*NC, normal ctrl; MCI, mild cognitive impairment; BMI, body mass index; TUGT, Timed Up and Go Test; MMSE, the Mini-Mental State Examination; IPAQ, International Physical Activity Questionnaire; HDL, high-density lipoprotein; LDL, low-density lipoprotein*.

### Alterations in serum metabolites profiling of the NC and MCI groups

**Figure 1:**
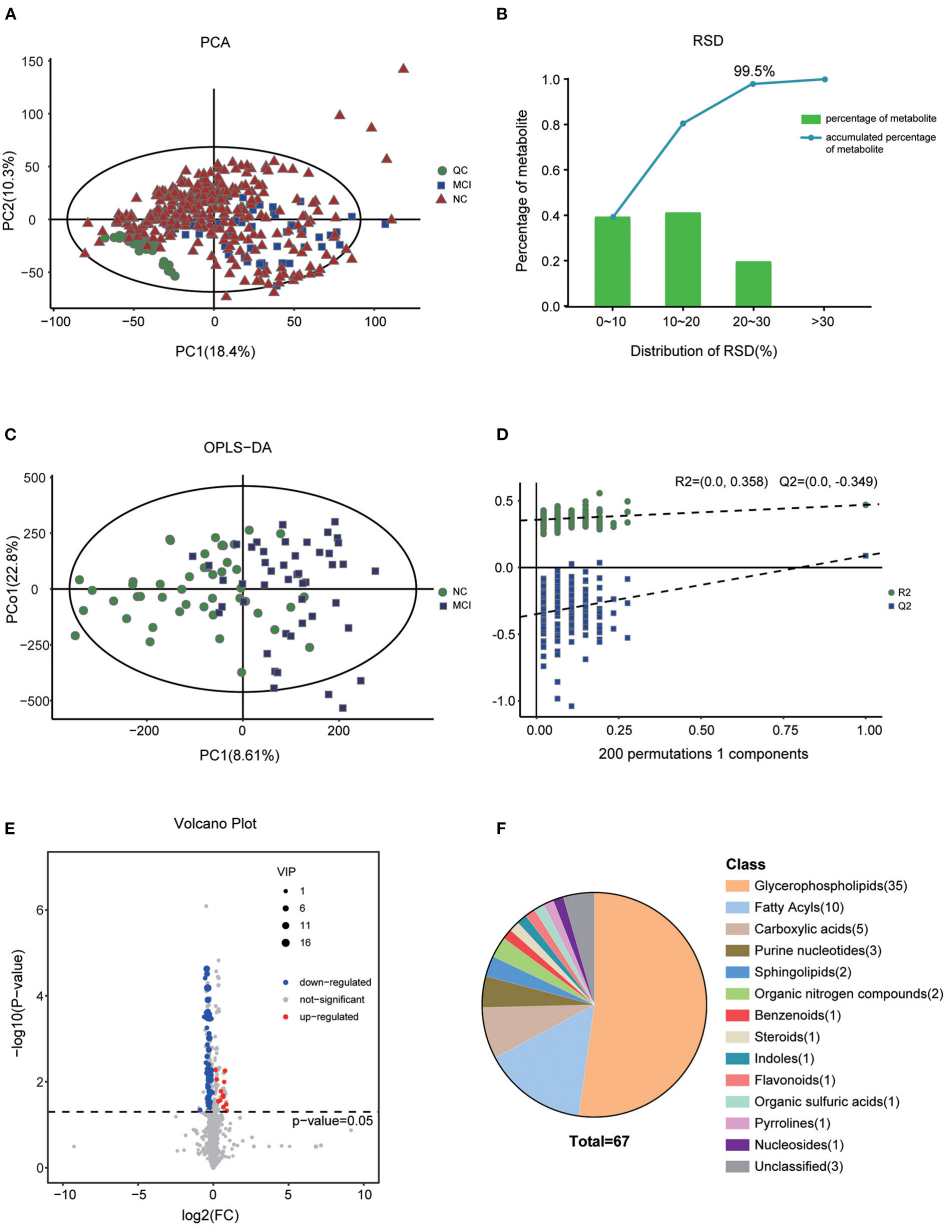
Altered metabolic profiles in MCI compared with NC. **(A)** Score plot of PCA in the cohort. **(B)** RSD distribution of metabolites in QC samples. **(C)** OPLS-DA score plot of MCI and the matched NC groups. **(D)** Statistical validation of the OPLS-DA model by permutation testing with 200 iterations. **(E)** Volcano plot of the differential metabolites filtered by the univariate analysis between the NC and MCI groups. **(F)** The pie chart shows the classification and number of significantly disturbed metabolites.

To illustrate the metabolic alterations, we performed an untargeted metabolomics approach using plasma samples. Totally, 4,488 features in ESI+ mode and 3,045 ion signatures in ESI- mode were detected, and 1,008 metabolites were identified. As shown in Figure 1A, the quality control (QC) samples clustered in the center of the principal component analysis (PCA) score plots suggesting that the analyses were repeatable and robust. Then, we further analyzed the relative SD (RSD) of metabolites in QC samples; 99.5% of the metabolites had RSD values of <30%, which further confirmed the reliability of the data (Figure 1B). To maximize the identification of differential metabolites between MCI and the matched NC groups, we constructed an OPLS-DA analysis on the metabolic spectrum and a tendency for separation can be observed (Figure 1C). The model was confirmed to not be overfitted following 200 permutation tests (Figure 1D). Among all the identified metabolites, 67 metabolites contributed significantly to the distinction of NC and MCI with VIP values of >1 and *p*-values of <0.05 (Figure 1E). The classification of the metabolites is shown in Figure 1F.

### Dysregulation of metabolic pathways in MCI

**Figure 2:**
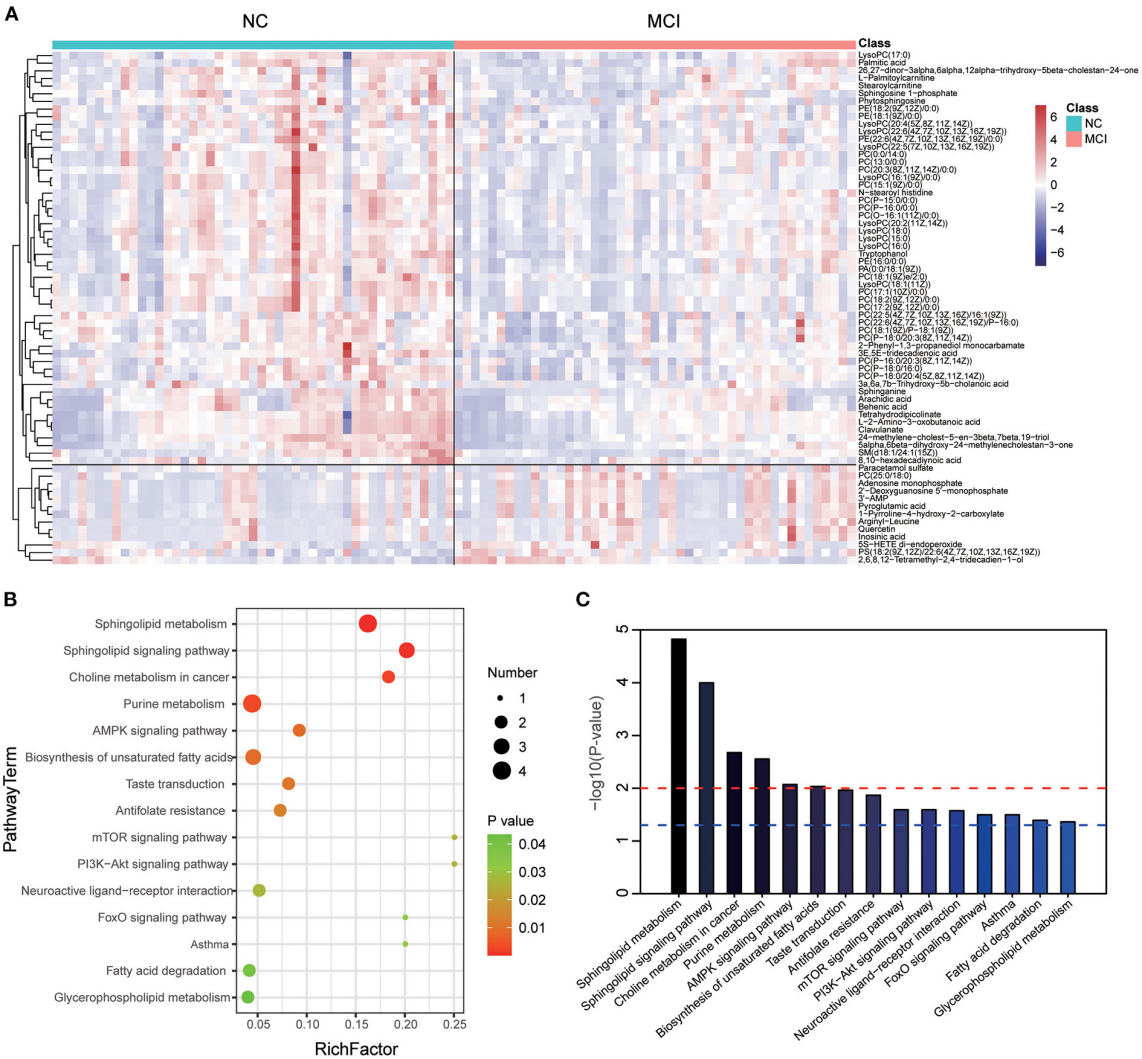
Heat map and metabolic pathway analysis based on the differentiated plasma metabolites. **(A)** The heat map showed the important differential metabolites profiling in serum samples of the NC and MCI groups. The blue color indicates a decreased level, and the red color indicates an increased level. **(B)** Pathway analysis indicates sphingolipid metabolism is the most statistically enriched pathway. **(C)** Plasma metabolite-based metabolic pathway analysis.

The levels of these metabolites were visualized by a heat map (Figure 2A), in which colors represent increased (red) or decreased (blue) abundance, with the intensity reflecting the corresponding concentration. Of these, levels of 54 metabolites were decreased in the MCI group (refers to the ratio of MCI/NC), including Phosphatidylcholines (PCs), Lysophosphatidylcholines (LysoPCs), Phosphatidylethanolamines (PEs), Phosphatidic Acids (PAs), Sphingomyelin (SM), Sphingosine-1-phosphate (S1P), Sa, fatty acids, fatty acid esters, fatty amides, and amino acids. Conversely, levels of 13 metabolites, including PS (18:2/22:6), PC (25:0/18:0), 5(S)-Hydroxy Eicosatetraenoic Acid (5S-HETE), purine nucleotides, and organic acids, were significantly increased in MCI. Next, we investigated which metabolic pathways may be behind the observed metabolic profile changes found to be associated with MCI. We performed pathway enrichment analysis and showed that sphingolipid metabolism, sphingolipid signaling pathway, choline metabolism in cancer, and purine metabolism were the most significantly perturbed pathways in MCI (Figures 2B,C). The characteristics indicated that dysregulation of sphingolipid metabolism may contribute to the occurrence of MCI.

### Evaluation of metabolites panel and combined with physical performance for the diagnosis of MCI

**Figure 3:**
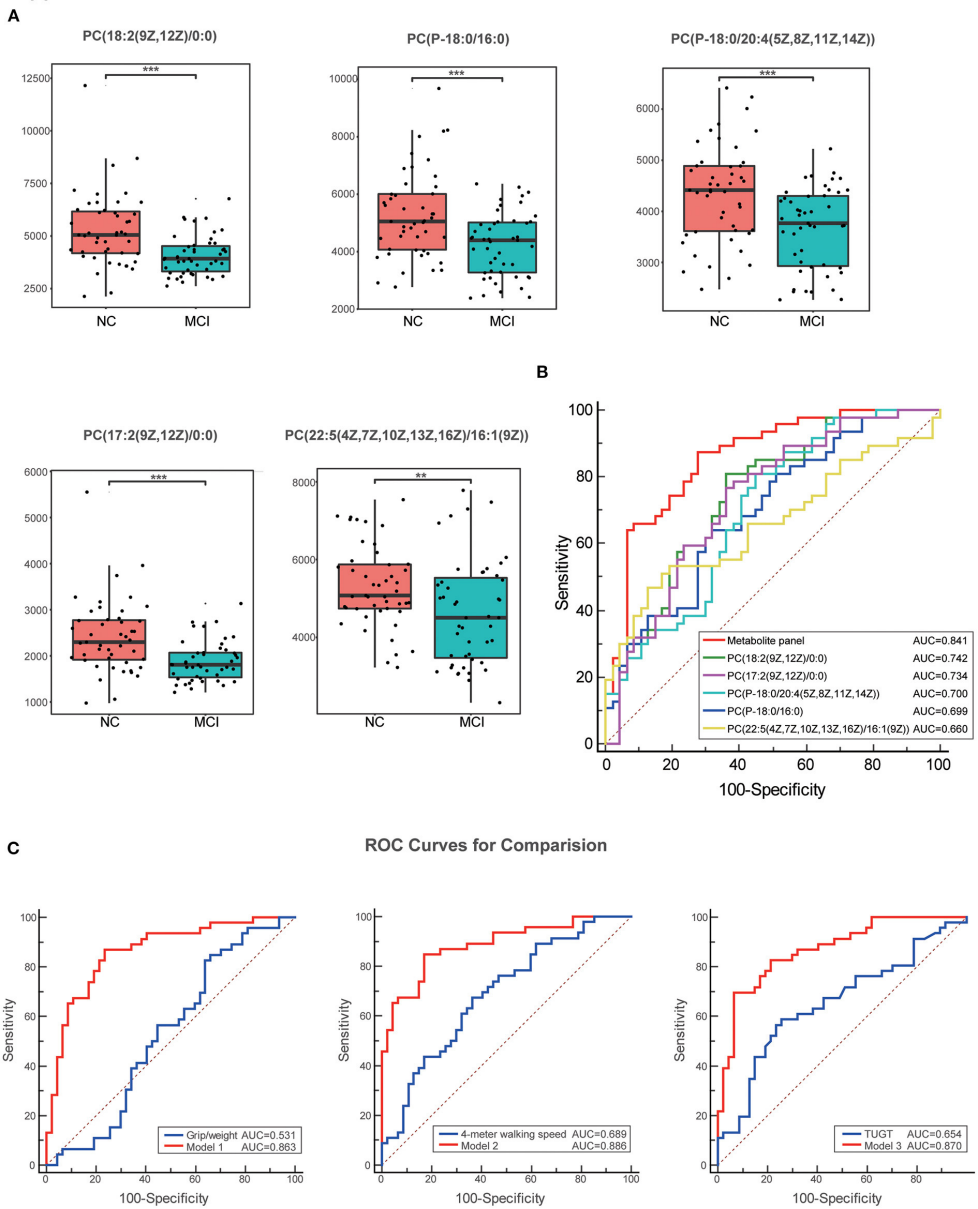
Box plots and the ROC curves for the most significantly varied differential metabolites. **(A)** Box plots show the significant changes in metabolite levels of the first five PCs. Data were expressed as means ± SE. ** indicates *p* < 0.01, *** indicates *p* < 0.001. **(B)** The ROC curves of the metabolite panel and each metabolite to discriminate MCI from the NC groups. **(C)** Comparison of the ROC curves for physical performance combined with metabolites with single physical performance tests. These combined metabolite panels showed satisfactory diagnostic performance for distinguishing MCI from NC. Model 1 includes grip/weight plus metabolites, the AUC = 0.863; model 2 includes walking speed plus metabolites, the AUC = 0.886; and model 3 includes TUGT plus metabolites, the AUC = 0.870. ROC, the receiver operating characteristic; TUGT, Timed Up and Go Test; MCI, mild cognitive impairment.

The VIP score identified the metabolites that contributed the most to the difference in metabolic profiles, and the top 20 metabolites are shown in Table 2. The result showed that PC levels in patients with MCI significantly contributed the most to the difference between the two groups and the significant changes in metabolite levels of the first five PCs are shown in Figure 3A. The ROC curves of classification models distinguishing MCI from NC using the 5 metabolites were plotted and the AUC values for each metabolite were 0.742, 0.734, 0.700, 0.699, and 0.660, respectively. The discriminant power was improved by combining the 5 metabolites using binary logistic regression analysis with the AUC value reaching 0.841 (Table 3 and Figure 3B). Then, the ROC curves using the metabolite panel and physical performance were plotted. The AUC values were as follows: metabolite panel, AUC = 0.841; grip/weight, AUC = 0.531; walking speed, AUC = 0.689; and TUGT, AUC = 0.654 (Table 3). These combined metabolite panels showed satisfactory diagnostic performance for distinguishing MCI from NC with the AUC value reaching 0.863, 0.886, and 0.870 (model 1 includes grip/weight plus metabolites, AUC = 0.863; model 2 includes walking speed plus metabolites, AUC = 0.886; model 3 includes TUGT plus metabolites, AUC = 0.870) (Figure 3C). Noticeably, these models were superior to that of each physical performance test alone in discriminating MCI (*P* < 0.05) (Table 4).

**Table 2:** The top 20 metabolites contributed most to the difference in metabolic profiles.

m/z	Ion mode	Metabolites	kegg	VIP	log2 (FC)
564.330	neg	PC (18:2(9Z,12Z)/0:0)		12.73	0.38
768.588	pos	PC (P-18:0/16:0)		11.10	0.32
510.355	pos	LysoPC (17:0)	C04230	9.30	0.19
794.604	pos	PC [P-18:0/20:4(5Z,8Z,11Z,14Z)]		8.86	0.24
480.309	neg	LysoPC (15:0)	C04230	8.67	0.20
566.346	neg	LysoPC (18:1(11Z))	C04230	8.26	0.44
504.309	neg	PC (17:2(9Z,12Z)/0:0)		8.20	0.36
235.092	pos	L-2-Amino-3-oxobutanoic acid	C03508	7.97	0.27
850.560	neg	PC [22:5(4Z,7Z,10Z,13Z,16Z)/16:1(9Z)]	C00157	7.68	0.19
540.330	neg	LysoPC (16:0)	C04230	5.84	0.36
857.675	neg	SM [d18:1/24:1(15Z)]	C00550	5.66	0.37
792.586	pos	PC [18:1(9Z)/P-18:1(9Z)]	C00157	5.64	0.30
506.325	neg	PC (17:1(10Z)/0:0)		5.13	0.42
588.331	neg	LysoPC [20:4(5Z,8Z,11Z,14Z)]	C04230	4.21	0.32
568.362	neg	LysoPC (18:0)	C04230	3.89	0.26
538.315	neg	LysoPC (16:1(9Z)/0:0)	C04230	3.77	0.41
818.602	pos	PC [P-18:0/20:3(8Z,11Z,14Z)]		3.55	0.21
612.331	neg	LysoPC [22:6(4Z,7Z,10Z,13Z,16Z,19Z)]	C04230	3.14	0.38
274.274	pos	Palmitic acid	C00249	3.04	0.23
476.278	neg	PE (18:2(9Z,12Z)/0:0)		2.99	0.29

*PC, Phosphatidylcholine; SM, Sphingomyelin; PE, Phosphatidylethanolamine; VIP, Variable Importance in the Projection; FC, Fold change*.

**Table 3:** The AUC values for individual or combined metabolites and physical performance tests.

Model	AUC	95%CI	SE
PC(18:2(9Z,12Z)/0:0)	0.742	0.641–0.826	0.0516
PC(17:2(9Z,12Z)/0:0)	0.734	0.633–0.820	0.0520
PC[P-18:0/20:4(5Z,8Z,11Z,14Z)]	0.700	0.597–0.790	0.0541
PC(P-18:0/16:0)	0.699	0.596–0.790	0.0536
PC[22:5(4Z,7Z,10Z,13Z,16Z)/16:1(9Z)]	0.660	0.555–0.754	0.0573
Metabolite panel	0.841	0.794–0.904	0.0380
Grip/weight	0.531	0.425–0.635	0.0616
Walking speed	0.689	0.585–0.781	0.0549
TUGT	0.654	0.548–0.750	0.0578
Model 1	0.863	0.776–0.926	0.0386
Model 2	0.886	0.804–0.943	0.0343
Model 3	0.870	0.784–0.931	0.0364

*Model 1: Grip/weight, plus metabolite panel*.

*Model 2: Walking speed, plus metabolite panel*.

*Model 3: TUGT, plus metabolite panel*.

**Table 4:** Pairwise comparison of the ROC curves.

Model	Difference between areas	95%CI	SE	Z statistic	***P*-value**
Model 1 vs. Grip/weight	0.332	0.192–0.472	0.072	4.642	<0.001
Model 2 vs. Walking speed	0.197	0.094–0.300	0.053	3.742	<0.001
Model 3 vs. TUGT	0.216	0.102–0.329	0.058	3.719	<0.001

*Model 1: Grip/weight, plus metabolite panel*.

*Model 2: Walking speed, plus metabolite panel*.

*Model 3: TUGT, plus metabolite panel*.

## Discussion

Our study presented a comprehensive metabolomic evaluation for MCI in Chinese community-dwelling older adults. We performed untargeted metabolomics of 1,008 identified metabolites on plasma samples from 380 participants who underwent physical performance tests and obtained 47 normal subjects who were matched by age, sex, BMI, and education to exclude the effect of confounding factors. We found that a series of metabolites were associated with MCI, and the dysregulation of sphingolipid metabolism was mostly related to the occurrence of MCI. Besides, we developed a discriminant model consisting of PCs to assist in the diagnosis of MCI. Based on the relationship we previously found between physical performance and MCI (Liu et al., 2021), we combined physical performance tests with these metabolites and found that the models significantly improved their distinguished ability for MCI.

Lipids are a major chemical group that contributes significantly to the functioning of the brain. Previous work has documented altered phospholipids concentrations in the brain in aging, cognitive decline, MCI and dementia (Grimm et al., 2011). Mapstone et al. (2014) reported a panel of plasma phospholipids, namely, lysoPCs and PCs, from peripheral blood that identified cognitively normal adults who would progress to either aMCI or AD within 2–3 years from those who remained cognitively normal. Their untargeted metabolomic and lipidomic profiling yielded 2,700 positive-mode features and 1,900 negative-mode features and revealed lower plasma levels of amino acids and PCs in converter participants who later converted to aMCI/AD. Our results have similarities to theirs; in our study, we used untargeted metabolomics, including 7,533 features in ESI+ mode and ESI- mode, and 1,008 metabolites were identified. We found that 67 metabolites, including PCs, LysoPCs, PEs, PSs, PAs, SMs, S1P, Sa, fatty acids, fatty acid esters, fatty amides, and amino acids, were different between groups. Among them, 54 metabolites, including PCs, LysoPCs, PEs, PAs, SM, S1P, Sa, fatty acids, fatty acid esters, fatty amides, and amino acids, were significantly decreased in MCI (Figure 2A). However, one study showed that phospholipids and metabolites were altered in MCI and dementia, but this cross-sectional association was relatively weak and did not improve the detection of MCI and dementia (Li et al., 2016). Our results reinforce the role of LysoPCs and PCs in the early identification of cognitive impairment.

Phosphatidylcholines (PCs), a fundamental brain phospholipid, are synthesized through the Kennedy pathway from three precursors: choline, pyrimidine, and polyunsaturated fatty acids (Moessinger et al., 2014; Zhang et al., 2021). Phospholipids are altered with dementia and some studies suggest their plasma levels may be useful in the detection of cognitive impairment. A decrease in PCs and lysoPCs in peripheral blood samples from patients with Alzheimer's disease has been previously reported (Gonzalez-Dominguez et al., 2014; Mapstone et al., 2014; Whiley et al., 2014). The reduced levels of PCs might be linked with the aberrant activity of phospholipase A2 (PLA2), which are enzymes that catalyze the cleavage of fatty acids from the sn-2 position of phospholipids, producing free fatty acids and LysoPCs. It has been reported that β-amyloid_42_ peptides that aggregate in the brain of patients with cognitive impairment increase the activity of PLA2 (Hicks et al., 2008). Some studies also reported altered LysoPC levels in Alzheimer's patient plasma (Li et al., 2010; Gonzalez-Dominguez et al., 2014). LysoPCs are the products of PLA2-catalyzed reactions that rapidly acylate with acetyl-CoA to maintain normal neuronal membrane composition. Several LysoPCs (including lysoPC a C18:1 and LysoPC a C18:2) have been reported to be increased (Grimm et al., 2011), but there was also a partial decrease in LysoPCs levels. The reasons may be that LysoPCs are not only glycerophospholipid metabolism intermediates but also serve as mediators in multiple neuronal pathways (Frisardi et al., 2011).

Besides, sphingolipids used to be recognized as the components of biological membrane and it is also associated with cognitive function. A study found a significant association of sphingomyelin (SM) with the prevalence of MCI and dementia (Li et al., 2016). Higher concentrations of SM(OH) C24:1 was significantly associated with a lower prevalence of dementia, and our results are consistent with literature findings on the link between cognitive impairment and decreased SM (d18:1/24:1) level. There is now clear evidence that sphingolipid catabolism is directly linked to neurodegenerative disorders in the brain (Haughey et al., 2010), and findings have identified pathways of the sphingolipid metabolism and decreased SM levels that contribute to Alzheimer's pathology (He et al., 2010; Mielke and Lyketsos, 2010). In our study, we observed that SM (d18:1/24:1) was significantly decreased in plasma of MCI, which indicates that this lipid metabolite might be useful as an additional diagnostic biomarker in MCI.

The National Institute on Aging and Alzheimer's Association has proposed a research framework in which cerebrospinal fluid (CSF) and imaging (magnetic resonance and PET)-based measures of Aβ (A), tau (T), and neurodegeneration (N) are incorporated into the ATN system. It can be used to reliably diagnose AD and identify MCI, with high diagnostic accuracy (Blennow et al., 2010; Olsson et al., 2016). Although the clinical diagnosis of MCI and AD is currently based on CSF or neuroimaging biomarkers, noninvasive and inexpensive blood-based biomarkers deserve to be investigated for initial screening of large sample populations. Kwangsik Nho et al. assessed the association of bile acid metabolites with the “A/T/N” (amyloid, tau, and neurodegeneration) biomarkers of AD and MCI, which was very enlightening (Nho et al., 2019). The correlation of serum PC levels and their relevant ratios with biomarkers of AD pathophysiology, including neuroimaging (MRI and PET) and CSF, may be a direction for our future research. In addition, our previous studies have reported that poor physical performance is significantly associated with MCI in community-dwelling older adults (Chen et al., 2021; Wu et al., 2021). Based on this, we expected to increase the ability to diagnose MCI early using physical performance tests combined with changes in plasma metabolites, and we developed a discriminant model consisting of PCs. Although previous studies have reported a few discriminant models derived from plasma, CSF, and urine (Kim et al., 2019; Weng et al., 2019; Zhang et al., 2019; Yilmaz et al., 2020), the advantage of our study is that the discriminant ability of the metabolites combined with walking speed model is as high as 0.886, which may have great potential for clinical applications in the future.

Our study has several advantages: (1) we have used an advanced high-resolution mass spectrometry metabolomics approach to capture up to seven thousand spectral signatures in human plasma and identify more than a thousand metabolites, which is unprecedented for most studies; (2) we used propensity score matching when selecting the control population for MCI, excluding the effects of confounders age, gender, BMI, and education; and (3) the AUC of screened metabolite panel of PCs was as high as 0.841 and the diagnostic ability of early screening MCI was further enhanced on the basis of physical performance tests, which is of great reference value for clinical application. There are some limitations of this study. First, although we matched age, sex, BMI, and education level in the NC and MCI groups, we did not consider diet and nutrition, which might influence cognitive function. Second, although our untargeted metabolomics can detect and identify 1,008 products, the sample size is limited, and we only test these metabolomes at one point; further prospective study will be more helpful in certifying the association of these metabolites with MCI dynamic changes. Third, the precise molecular mechanisms underlying the results are still unknown, we were unable to evaluate the role of metabolites such as PCs in cognitive function, and mechanistic studies are needed to clarify the exact role of these metabolites in MCI. Finally, this study is based on a cross-sectional design. Future studies will need to increase the sample size and perform follow-ups in this population to further verify the models.

## Conclusion

In conclusion, our results suggest that several sphingolipid metabolites are associated with MCI and that a panel of metabolites of PCs might be highly useful as a novel plasma biomarker for the diagnosis of MCI. Physical performance tests combined with metabolites tend to be more sensitive for the identification of MCI; further longitudinal studies and reproduction will be necessary to introduce metabolomic markers of PCs into clinical applications.

## Data availability statement

The raw data supporting the conclusions of this article will be made available by the authors, without undue reservation.

## Ethics statement

The studies involving human participants were reviewed and approved by Shanghai University of Medicine and Health Science. The patients/participants provided their written informed consent to participate in this study.

## Author contributions

YZ and PH conceived the concept and design of the study. PS, XC, XY, and HZ contributed to data collection, data entry, and data cleaning. PH, CF, and FX contributed to the data analysis and interpretation of the study results. YZ and PS drafted the article or revised it critically for important intellectual content. QG provided administrative support and contributed to the acquisition of funding. All authors have approved the final version of the manuscript.

## Funding

This work was supported by the National Natural Science Foundation of China (No. 82172552).

## Conflict of interest

The authors declare that the research was conducted in the absence of any commercial or financial relationships that could be construed as a potential conflict of interest.

## Publisher's note

All claims expressed in this article are solely those of the authors and do not necessarily represent those of their affiliated organizations, or those of the publisher, the editors and the reviewers. Any product that may be evaluated in this article, or claim that may be made by its manufacturer, is not guaranteed or endorsed by the publisher.
